# Effects of UV Aging on Antimicrobial Performance and Color Stability of Hygienic Additive-Modified Polyurethane and Waterborne Coatings Applied to Oriental Beech (*Fagus orientalis* L.)

**DOI:** 10.3390/polym18080937

**Published:** 2026-04-11

**Authors:** Hacı İsmail Kesik, Recep Aykan, Perihan Akbaş

**Affiliations:** 1 Department of Wood Products Industrial Engineering, Faculty of Technology, Gazi University, 06560 Ankara, Türkiye; 2 Forestry Department, Ayancık Vocational School, Sinop University, 57000 Sinop, Türkiye; raykan@sinop.edu.tr; 3 Department of Medical Services and Techniques, Vocational School of Health Services Samsun, Ondokuz Mayıs University, 55270 Samsun, Türkiye; perihan.akbas@omu.edu.tr

**Keywords:** Ag-modified coating, waterborne coating, polyurethane coating, antimicrobial activity, color stability

## Abstract

This study was carried out to investigate the antimicrobial performance and color stability of silver (Ag)-modified polyurethane and waterborne coating systems applied to Oriental beech *(Fagus orientalis* L.) wood after the specimens were subjected to UV aging for 24 h. Antimicrobial activity and color stability were evaluated before and after aging against *Escherichia coli* (*E. coli*, ATCC 25922), *Staphylococcus aureus* (*S. aureus*, NCTC 13552), and *Candida albicans* (*C. albicans*) in accordance with the JIS Z 2801 standard. Color changes were determined using CIELab parameters (ΔL*, Δa*, Δb*, and ΔE*) in accordance with the TS EN ISO 16474-3 standard. Prior to UV exposure, the highest antibacterial activity against *E. coli* occurred in Ag-modified waterborne varnish coatings, whereas the highest antifungal activity against *C. albicans* occurred in Ag-modified polyurethane paint systems. After UV aging, antimicrobial performance varied depending on the coating type. Particularly, Ag-modified waterborne varnish coatings retained significant antibacterial activity against *E. coli* and *S. aureus* and exhibited the highest antifungal performance against *C. albicans*. Color analysis revealed that UV exposure also caused significant changes in all coating systems. The most pronounced variations were observed for the lightness difference (ΔL*), red–green color difference (Δa*), and yellow–blue color difference (Δb*) parameters, while the lowest total color difference (ΔE*) values were observed for Ag-modified polyurethane and Ag-modified waterborne varnish coatings. Overall, Ag-modified waterborne varnish systems demonstrated superior performance in both antimicrobial activity and color stability after UV aging.

## 1. Introduction

Wood is widely used in interior and furniture applications due to its renewable nature [1], relatively low energy requirement during production [2], and its aesthetic and functional advantages [3]. In particular, Oriental beech (*Fagus orientalis* L.) stands out as a preferred species owing to its homogeneous fiber structure and high machinability. However, due to its lignocellulosic composition, wood material is highly susceptible to environmental factors. Ultraviolet (UV) radiation is known to induce photo-oxidative reactions in lignin, leading to discoloration on the surface [4]. Additionally, fluctuations in moisture create favorable conditions for microbial growth, thereby accelerating biological degradation processes [5]. These degradation mechanisms not only impair the visual quality of wood surfaces but also adversely affect their long-term durability and service life.

To mitigate these adverse effects, coating systems have been widely developed and are considered a fundamental approach for protecting wood surfaces [6]. Coatings can partially block UV radiation, thereby reducing color changes, and may suppress microbial growth through the incorporation of functional additives [7]. In this context, waterborne coatings are recognized for their environmentally friendly characteristics [8], whereas polyurethane-based coatings are known for their superior mechanical durability and surface performance [9]. These characteristics indicate that coating systems should be regarded not only as passive protective layers but also as multifunctional materials capable of enhancing both durability and hygienic performance.

It is well established that extractive compounds present in wood materials can exhibit antibacterial properties to a certain extent [10]. However, the effect of these natural antimicrobial properties is not sufficient during the practical use of wood material. [11]. Furthermore, the relatively limited number of studies in this field highlights the need for more comprehensive and systematic research [12]. This limitation necessitates the development of additional strategies to effectively control microbial growth on wood surfaces. In this context, antimicrobial coatings have emerged as an effective approach, particularly for frequently contacted surfaces, by inhibiting the adhesion and proliferation of microorganisms [13].

Microorganisms such as *E. coli*, *S. aureus*, and *C. albicans* are commonly encountered on surfaces and can adhere, survive, and proliferate under suitable conditions, increasing the risk of infection [14,15]. This indicates that coating systems are considered not only physical protective barriers but also active hygienic surfaces. In this study, the term “hygienic additives” refers to antimicrobial agents incorporated into coating formulations to inhibit microbial growth. Although antimicrobial additives can enhance this functionality, several studies have reported that their effectiveness strongly depends on the type of additive and coating formulation [16,17]. This indicates that the presence of additives alone is not sufficient, and their interaction within the coating matrix plays a critical role.

In the absence of functional additives, pure polymer coatings generally exhibit limited protective performance, necessitating the incorporation of active components [18]. Previous studies have demonstrated that metal oxide-based additives such as ZnO can improve UV resistance [19], while Ag-based additives are particularly effective in enhancing antibacterial performance [20]. It has also been reported that waterborne coatings modified with high Ag content exhibit enhanced antibacterial performance and provide long-term protection [21]. In addition, inorganic and carbon-based nanomaterials such as SiO_2_ and graphene can improve coating performance by forming barrier effects on the surface [22,23]. Furthermore, nanopigments have been shown to enhance color stability due to their UV-absorbing properties [24,25]. However, the effectiveness of these additives is governed by their dispersion within the matrix and their physicochemical stability under environmental stress conditions. This is supported by studies indicating that antimicrobial performance in nano-Ag-modified coatings varies depending on material type and that UV aging can reduce antibacterial effectiveness [26]. These findings suggest that additive incorporation does not always guarantee enhanced performance and may, under certain conditions, lead to functional deterioration.

The performance of coating systems is not determined solely by their initial properties but is strongly influenced by their stability under environmental conditions. UV exposure, in particular, can induce chemical changes in coating surfaces, affecting both optical properties and antimicrobial activity. Studies have shown that insufficient photoactive components limit the formation of reactive species, thereby reducing antimicrobial effectiveness [27,28]. Similarly, the photocatalytic activity of coatings depends on their composition and surface characteristics [29,30]. Conversely, some studies have demonstrated that UV exposure can enhance antimicrobial performance by promoting the migration of active components to the surface and increasing the formation of reactive oxygen species (ROS) [31]. Therefore, UV-induced processes should be considered dual-effect mechanisms that may either enhance or deteriorate coating performance, depending on system composition.

The gloss, as a key aesthetic parameter of transparent coatings, is affected by the coating–substrate interaction and environmental conditions, which may influence its stability over time [32]. In addition, UV radiation is one of the primary environmental factors affecting the aesthetic properties of coating systems, particularly by triggering photo-oxidative degradation processes in wood components. These processes lead to chemical alterations in lignin and hemicellulose within the wood structure, causing visible color changes on the surface. The investigation of such color changes, including darkening and yellowing observed in coated wood surfaces, and the evaluation of their effects are of critical importance for assessing the visual performance of coating systems [33,34].

In this context, color stability is a key parameter for evaluating the aesthetic performance of coating systems under environmental exposure. Colorimetric parameters (L*, a*, b*) and the ΔE* are widely used to quantify surface color changes and are influenced by both coating type and substrate properties [33]. UV radiation is known to cause darkening (ΔL*) and yellowing (Δb*) on coated wood surfaces as a result of lignin photodegradation. In contrast, systems containing pigments and nanoparticles may exhibit improved color stability by partially blocking UV radiation. Moreover, functional additives with UV-absorbing or photocatalytic properties can further reduce color changes. However, the effectiveness of these additives largely depends on the coating formulation and environmental conditions, highlighting the importance of systematic evaluation under UV aging [19].

Despite these findings, the behavior of antimicrobial additives in coating systems exposed to UV radiation has not yet been clearly and consistently established in the literature. Existing studies indicate that UV irradiation can enhance antimicrobial activity in certain coating systems, whereas in others it may cause photodegradation that reduces antimicrobial efficacy. Similarly, the effects on color stability have been reported to vary depending on the coating type, the binder matrix, and the chemical nature of the incorporated additives. This variability limits the comparability of existing findings and hinders the development of standardized and predictive evaluation approaches for coating performance under UV exposure [28,31].

Although a growing body of research exists, most studies have addressed UV resistance, color stability, and antimicrobial performance separately, indicating a significant gap in the literature. In this context, this study provides a novel approach by simultaneously evaluating both biological (antimicrobial) and aesthetic (color stability) performance under UV aging conditions. Studies that simultaneously evaluate the relationship between biological functionality and aesthetic durability under UV aging conditions remain limited. In particular, studies evaluating the combined biological and aesthetic performance of water-based and polyurethane coating systems modified with hygienic additives under UV-aging conditions when applied to beech wood are scarce.

The hypothesize of this study was coating type and the presence of hygienic additives significantly influence both antimicrobial performance and color stability after UV aging conditions. Accordingly, the aim of this study was to compare the antimicrobial activity and color stability of water-based and polyurethane coating systems containing hygienic additives applied to Oriental beech after UV aging and to elucidate the interaction between antimicrobial performance and color stability with respect to coating composition and UV-induced effects.

## 2. Materials and Methods

### 2.1. Wooden Material

In the study, Oriental beech *(Fagus orientalis* L.), which is widely used in the furniture and toy industries in Türkiye, was chosen as the experimental material. The beech wood was supplied by Ayancık Ders Araçları Company (Ayancık, Turkey). Test specimens were randomly prepared from first-grade lumber characterized by straight grain structure and the absence of knots, cracks, and visible defects, as well as minimal variation in color and density.

Some physical and mechanical properties of the wood material were tested in accordance with the procedures described in TS ISO 13061-1 (2021) [35], TS ISO 13061-2 (2021) [36], TSE EN 322 (1999) [37], and TSE EN 323 (1999) [38]. The average moisture content (MC) value was 7.99%, and the density value was 0.65 g/cm^3^ for beech.

### 2.2. Protective Surface Coating Materials

In this study, polyurethane and waterborne varnishes that are commonly used in the furniture industry in Türkiye were selected as surface coating materials. To obtain colored coatings, a red nano-sized pigment was incorporated into the varnish formulations. The polyurethane and waterborne varnishes were supplied by Kubilay Boya Company (Izmir, Turkey), while the nano-color pigment was obtained from Kimetsan Company (Ankara, Turkey). Four groups of manufacturer-supplied varnishes were modified by adding 5% red nano pigment, resulting in eight coating formulations in total. Within the scope of this study, the coating systems examined were classified as follows: waterborne varnish (WBV), Ag-modified waterborne varnish (HWBV), waterborne paint (WBP), Ag-modified waterborne paint (HWBP), polyurethane varnish (PUV), Ag-modified polyurethane varnish (HPUV), polyurethane paint (PUP), and Ag-modified polyurethane paint (HPUP). Selected technical properties and application parameters of the varnish and paint systems are presented in Table 1.

The hygienic performance of the coating systems was achieved through a commercially supplied antimicrobial component based on inorganic Ag nanoparticles. This component was not introduced separately during the experimental procedure; rather, it had already been incorporated into the coating formulations by the manufacturer. Owing to patent and proprietary limitations, the detailed chemical composition of the additive cannot be disclosed. Nevertheless, the materials were used in the form provided, and all applications were carried out in accordance with the manufacturer’s recommended procedures.

The antimicrobial mechanism of Ag-based additives primarily involves the release of Ag^+^ ions, which interact with microbial cell membranes and cause structural damage. This process leads to increased membrane permeability and disruption of cellular integrity. In addition, Ag^+^ ions can penetrate cells and interact with thiol (–SH) groups of proteins, thereby inhibiting enzymatic systems and disrupting cellular metabolism. Furthermore, Ag nanoparticles are known to induce the formation of ROS, which contribute to oxidative stress and cellular damage, ultimately resulting in microbial inactivation [40].

In addition to the antimicrobial additive, nanopigments were incorporated into the selected coating systems at a concentration of 5%, in a water-dispersed form. The pigment used in this study is a nano-engineered organic nanopigment applied to provide both coloration and functional properties to the coating system.

These nanopigments absorb and/or scatter UV radiation, thereby reducing photodegradation of the polymer matrix and improving color stability. Moreover, by acting as a UV barrier, they may indirectly protect antimicrobial additives against photodegradation. The type and commercial source of the coatings and additives used in this study were provided by the manufacturer.

The nanoscale structure of the additive increases its surface area, thereby facilitating more effective interactions with microorganisms. However, its performance in coating systems depends not only on its intrinsic properties but also on its dispersion within the matrix and its ability to migrate to the coating surface. These factors play a critical role in the emergence and sustainability of antimicrobial activity.

### 2.3. Preparation of the Test Specimens

In this study, a total of 48 specimens were prepared. Of these, 16 specimens with dimensions of 320 × 80 × 10 mm were produced for antimicrobial activity tests, while the remaining 32 specimens, with the same dimensions, were prepared for color measurements. The specimens consisted of Oriental beech wood substrates combined with eight different protective coating systems (paints and varnishes). Separate sets of specimens were used for antimicrobial and color analyses to avoid potential interference between measurements. From each specimen, 10 measurements were taken for antimicrobial activity evaluation and 10 for color analysis. A total of 160 measurements were obtained for each test.

After cutting the specimens to precise dimensions, the surfaces were sequentially sanded with 180- and 220-grit sandpaper prior to coating. Surface dust was then removed using a soft-bristle brush and a vacuum cleaner. Coating application was carried out in accordance with the manufacturer’s recommendations and the principles of TS EN ISO 28199-1 (2021) [41]. The coatings were applied in two layers using a spray gun at room temperature (20 °C).

**Figure 1:**
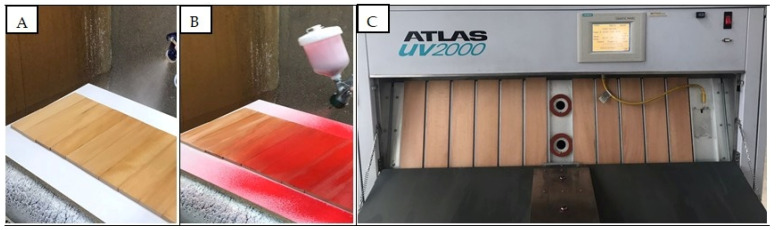
Images of varnish (**A**) and paint (**B**) applications and UV aging process (**C**).

The coated specimens were allowed to dry for three weeks at room temperature while positioned parallel to the ground and protected from direct sunlight. Subsequently, all test specimens were conditioned in a climate chamber at 20 ± 2 °C and 50 ± 3% relative humidity until constant mass was achieved. Of the specimens that reached constant mass, 36 specimens were subjected to UV aging for 24 h using UVA-340 lamps in an Atlas UV aging device (Atlas, Mount Prospect, IL, USA) in accordance with TS EN ISO 16474-3 (2021) [42]. Varnish and paint applications and the UV aging process on the specimens’ surfaces are shown in Figure 1.

**Figure 2:**
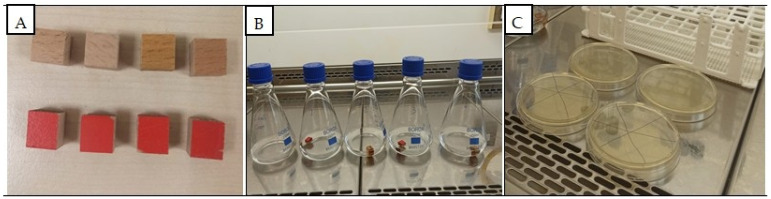
Images of the specimens for antimicrobial activity tests (**A**), preparation for activities (**B**) and applications (**C**).

For antimicrobial testing, 8 specimens were taken from each of the control and UV-aged groups and cut to dimensions of 10 × 10 × 10 mm, resulting in a total of 160 specimens prepared for antimicrobial activity evaluation. The specimens prepared for antimicrobial activity tests, preparations for activities, and applications are given in Figure 2.

### 2.4. Antimicrobial Activity and Analyses

Antimicrobial activity tests are commonly applied to water-insoluble materials such as textiles, plastics, and wood. In this study, a modified version of the JIS Z 2801 [43] standard was employed to evaluate the antimicrobial activity of beech wood specimens coated with protective coatings. Since they are frequently encountered in domestic environments, *E. coli*, *S. aureus*, and *C. albicans* were selected as test microorganisms. Suspensions of *E. coli, S. aureus*, and *C*. *albicans* were prepared in a total volume of 10 mL at a concentration of 5 × 10^5^ colony-forming units per milliliter (CFU/mL); test specimens measuring 1 cm^3^ were then introduced into these suspensions. Microbial suspensions prepared without test specimens at the same concentration (5 × 10^5^ CFU/mL) were used as controls. The tubes containing the specimens were shaken at 150 rpm for 4 h.

To detach microorganisms adhering to the specimen surfaces, both specimen-containing and control tubes were placed in an ultrasonic water bath for 30 s, followed by vortex mixing. Subsequently, 100 µL of the resulting suspension was transferred into 900 µL of physiological saline solution, and serial dilutions were prepared up to six levels. Petri dishes containing MHA were divided into six sections, and 25 µL of each dilution was inoculated onto the agar surface. After incubation at 37 °C for 24 h, antimicrobial activity was determined by counting CFU and was expressed as log10 and percentage reductions.

### 2.5. Measuring the Color Changing Properties

Color measurements, based on the CIELAB color system defined by the International Commission on Illumination (CIE), were performed using a Konica Minolta CM-2600d spectrophotometer (Konica Minolta, Tokyo, Japan) with a D65/10° illuminator/observer. The CIELab color space (L*, a*, b*) and the ΔE* parameters are internationally accepted methods for the quantitative evaluation of color changes. In this context, UV-aging tests are used as a reliable, accelerated method to predict the long-term aesthetic performance of coating systems.

When the specimens are exposed to environmental factors (such as UV-aging), the differences between measurements taken before and after the process are calculated to quantify color change. These differences (ΔL*, Δa*, Δb*) indicate the direction and magnitude of color change on the surfaces and serve as fundamental parameters for calculating the ΔE*. In this study, 10 measurements were performed for each specimen to obtain average values for both the control group and UV-aged specimens. Accordingly, the color difference parameters were calculated using the following equations (ISO 11664-4, 2008) [44].

## 3. Results and Discussions

Standard methods applicable to water-insoluble surfaces were employed in antimicrobial activity tests. Within the scope of the study, the antimicrobial activities of all specimens were evaluated under identical experimental conditions. The difference in microbial growth between the positive control and cultures treated with coating systems containing hygiene additives and nanopigments was assessed as logarithmic reduction (log reduction).

Color measurement tests were conducted following standard colorimetric methods suitable for opaque and semi-opaque surfaces such as wood. In this study, color measurements of all specimens were performed under identical conditions using the same instrument settings. Color data obtained before and after coating application and after UV aging were evaluated in the CIELAB color space. The ΔL*, Δa*, Δb*, and ΔE* parameters were calculated. In this way, color changes occurring between the control specimens and the surfaces treated with coating systems containing hygiene additives and nanopigments were quantitatively compared.

### 3.1. Results of Antimicrobial Activity Tests

#### 3.1.1. Antimicrobial Activity Against *E. coli*

**Figure 3:**
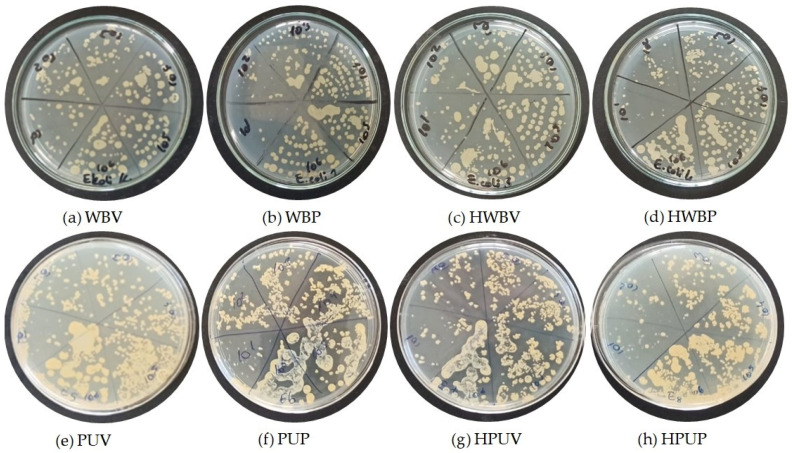
Illustrations of the colony growth images corresponding to the inoculation of *E. coli* dilutions on MHA medium.

Figure 3 presents images of colony growth obtained from *E. coli* dilutions inoculated onto MHA for selected coated specimens prior to UV aging. The specimens are coded as: (a) WBV, (b) WBP, (c) HWBV, (d) HWBP, (e) PUV, (f) PUP, (g) HPUV, and (h) HPUP.

**Figure 4:**
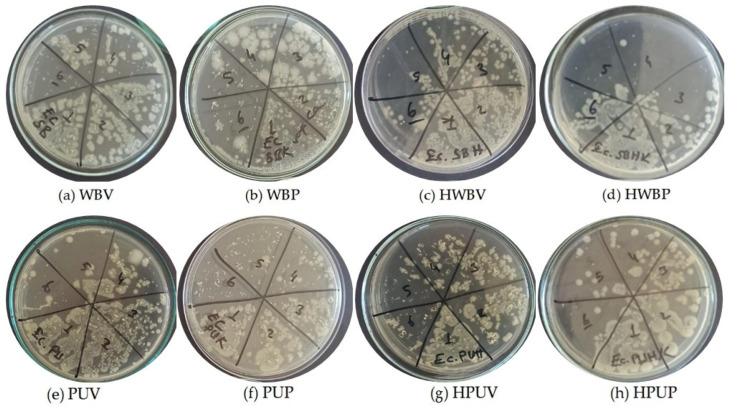
Images of colony growth obtained from *E. coli* dilutions inoculated onto MHA after UV treatment.

Figure 4 presents images of colony growth obtained from *E. coli* MHA following UV treatment of specimens coded as (a) WBV, (b) WBP, (c) HWBV, (d) HWBP, (e) PUV, (f) PUP, (g) HPUV, and (h) HPUP. In the figure, label 1 represents a bacterial dilution of 10^6^ CFU/mL, while label 6 corresponds to a dilution of 10^1^ CFU/mL.

Table 2 presents the comparative antimicrobial activity against *E. coli* of test specimens with protective coatings before and after UV aging, based on evaluations conducted in accordance with the JIS Z 2801 standard.

According to Table 2, the specimens coated with HWBP and subjected to UV aging exhibited a 96.2% reduction in *E. coli*, representing the highest antimicrobial activity observed in this study. Prior to UV aging, bacterial reductions of 75% and 26% were observed for the WBP- and HPUV-coated specimens, respectively; however, these values decreased after UV aging. This finding suggests that UV aging may adversely affect the antimicrobial performance of certain coating systems, likely due to the degradation of active components within the coating matrix, as also reported in previous studies [45].

In contrast, no measurable antimicrobial activity against *E. coli* was detected in the WBV, PUV, and HPUP coated specimens either before or after UV aging (log_10_ reduction = 0). This behavior can be explained by the limited surface availability and restricted mobility of antimicrobial agents within dense polymer matrices, particularly in polyurethane-based systems, which may hinder effective microorganism–surface interactions [46]. UV exposure did not significantly alter this condition, indicating a lack of UV-responsive mechanisms in these systems.

Among the water-borne systems, the most pronounced change was observed in the WBP specimen. While this system exhibited 75% antimicrobial efficacy prior to UV exposure, its activity decreased to 39.8% after 24 h of UV aging. This decrease suggests that UV radiation may partially degrade active components within the paint matrix, leading to a reduction in antimicrobial effectiveness, which is consistent with photodegradation mechanisms reported in the literature [21].

Conversely, the HWBV specimen showed a 74.3% increase in activity after UV exposure. This enhancement may be associated with UV-induced migration of active components toward the surface and/or photochemical activation processes, which can increase the availability of antimicrobial agents at the interface [31]. This behavior indicates that certain waterborne systems may exhibit UV-activated antimicrobial responses, in contrast to systems dominated by photodegradation.

Similarly, the HWBP specimen exhibited an increase in antimicrobial performance from 90% to 96.2% after UV aging, corresponding to an approximately 6.9% improvement. This observation suggests that the hygienic additives incorporated into this system possess relatively high UV resistance and photostability and may benefit from UV-induced activation mechanisms, depending on their chemical structure and interaction with the binder matrix.

For the polyurethane-based systems, no change in antimicrobial activity was observed for the PUV and HPUP specimens. This behavior could be attributed to the immobilization of active components within the highly crosslinked and dense polyurethane matrix, which limits their migration to the surface and reduces effective interaction with microorganisms. Therefore, UV exposure does not significantly activate or degrade antimicrobial agents in these systems.

In the case of the PUP specimen, antimicrobial activity emerged after UV aging, which was not observed prior to UV exposure. This behavior may be attributed to UV-induced formation of oxygen-containing functional groups (e.g., hydroxyl or carbonyl groups), which increase surface energy and promote microorganism–surface interactions, as reported for UV-modified polymer surfaces [27].

Conversely, the HPUV specimen showed a complete loss of activity, decreasing from 26% to 0%. This result indicates that UV radiation likely induced photodegradation of the active hygienic additives, leading to a complete loss of antimicrobial effectiveness, particularly in systems with limited photostability. Overall, these findings demonstrate that polyurethane-based systems are more prone to antimicrobial performance loss under UV exposure, whereas certain waterborne systems may exhibit either stability or activation depending on their formulation.

#### 3.1.2. Antimicrobial Activities Against *S. aureus*

Logarithmic and percentage comparisons of the antimicrobial activities of wood specimens against *S. aureus* before and after UV treatment are shown in Table 3.

According to Table 3, the HWBV coating system stands out as the material that maintained its activity against *S. aureus* after UV aging and exhibited an approximately 80% increase in activity compared with other systems. In the PUV and PUP specimens, the initially low antimicrobial activity increased to 60–70% after UV aging. In contrast, the antimicrobial activity that was initially observed at low levels in the HWBP- and HPUV-coated wood specimens completely disappeared following UV aging. No antimicrobial activity against *S. aureus* was detected in the remaining coating systems, and UV aging did not significantly change antimicrobial effectiveness in these specimens. Overall, these findings indicate that the response of coating systems to UV exposure is highly system-dependent, with some formulations exhibiting activation while others undergo deactivation.

Previous studies have reported that the antibacterial performance of Ag-modified coatings may vary depending on additive concentration and environmental conditions [21], and that such coatings can exhibit high antimicrobial activity against *S. aureus* [47]. The findings of the present study are in partial agreement with the literature, indicating that antimicrobial performance is strongly influenced by coating type, matrix structure, and UV exposure conditions.

In particular, the enhanced activity observed in certain systems may be associated with UV-induced activation mechanisms, such as increased surface availability or activation of antimicrobial agents, whereas the loss of activity in others can be attributed to photodegradation of active components or to their restricted mobility (immobilization) within the coating matrix. These contrasting behaviors highlight the dual role of UV exposure as both an activating and degrading factor in antimicrobial coating performance.

**Figure 5:**
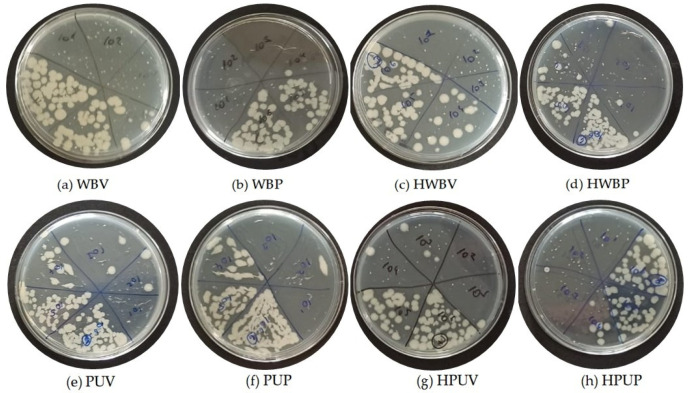
Images of colony development from inoculations of *S. aureus* dilutions onto MHA medium prior to UV treatment of specimens.

Figure 5 illustrates the colony growth obtained from *S. aureus* dilutions prior to UV exposure for the coded specimens. The images show bacterial colonies formed on MHA medium at different dilution levels, ranging from 10^6^ CFU/mL (Specimen (a), WBV) to 10^1^ CFU/mL (Specimen (f), PUP).

**Figure 6:**
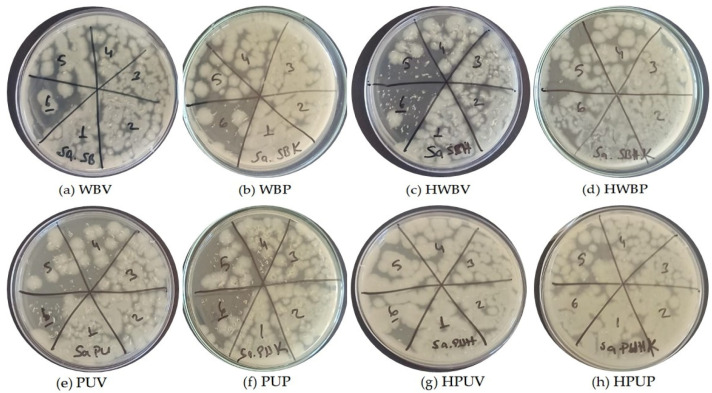
Images of colony development from inoculations of *S. aureus* dilutions from specimens after UV treatment onto MHA medium.

Figure 6 presents colony development images, obtained from *S. aureus* dilutions inoculated onto MHA medium prior to UV treatment, for the specimens coded as (a) WBV, (b) WBP, (c) HWBV, (d) HWBP, (e) PUV, (f) PUP, (g) HPUV, and (h) HPUP. In the figure, label 1 corresponds to a bacterial dilution of 10^6^ CFU/mL, whereas label 6 corresponds to a bacterial dilution of 10^1^ CFU/mL.

In antimicrobial tests conducted with Gram-positive bacteria, water-based coating systems generally exhibited low initial activity. However, antimicrobial efficacy increased by approximately 80% in the UV-aged HWBV specimen. This enhancement may be attributed to UV-induced migration of active components toward the surface and/or photochemical activation processes in coatings containing hygienic additives, leading to increased antimicrobial effectiveness [31].

In contrast, in the HWBP specimen, antimicrobial activity decreased from 8.8% to zero after UV aging. Similarly, in the HPUV system, the activity decreased from 18.7% to 0%, indicating a complete loss of antimicrobial effectiveness. This reduction may be associated with UV-induced photodegradation of certain hygienic additives or decreased surface availability, which limits their antimicrobial functionality, as reported for UV-sensitive antimicrobial systems [45].

On the other hand, a significant increase in antimicrobial activity was observed in UV-aged specimens of PUV (from 29.2% to 69.8%) and PUP (from 8.8% to 65.3%). This increase may be related to UV-induced oxidative modifications on polyurethane coating surfaces, resulting in the formation of hydrophilic functional groups (e.g., hydroxyl and carbonyl groups), which increase surface energy and reactivity. Such modifications can enhance interactions between bacterial cells and the coating surface, potentially leading to membrane damage and subsequent microbial inactivation [27]. Overall, these findings demonstrate that UV exposure can induce both activation and degradation mechanisms in antimicrobial coatings, depending on coating composition and additive stability.

#### 3.1.3. Antimicrobial Activities Against *C. albicans*

The antimicrobial activities of all wood specimens against *C. albicans* before and after UV treatment are presented in Table 4.

**Figure 7:**
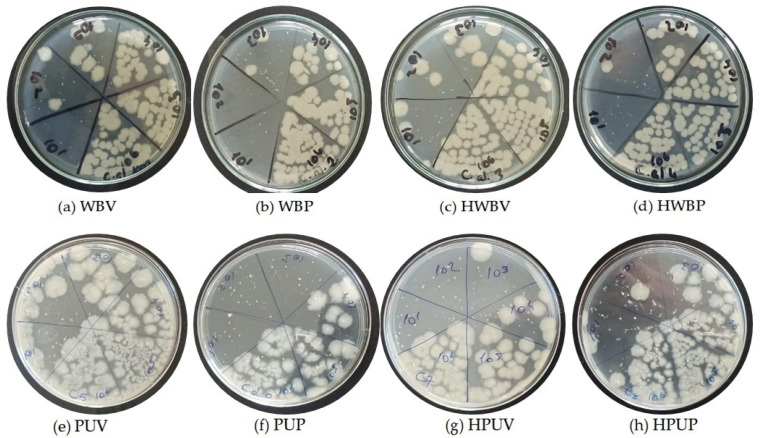
Images of colony development from inoculations of *C. albicans* dilutions onto MHA medium prior to UV treatment of the specimens.

Figure 7 presents the specimens coded as (a) WBV, (b) WBP, (c) HWBV, (d) HWBP, (e) PUV, (f) PUP, (g) HPUV, and (h) HPUP prior to UV treatment. In the figure, label 1 corresponds to a bacterial dilution of 10^6^ CFU/mL, whereas label 6 represents a dilution level of 10^1^ CFU/mL.

**Figure 8:**
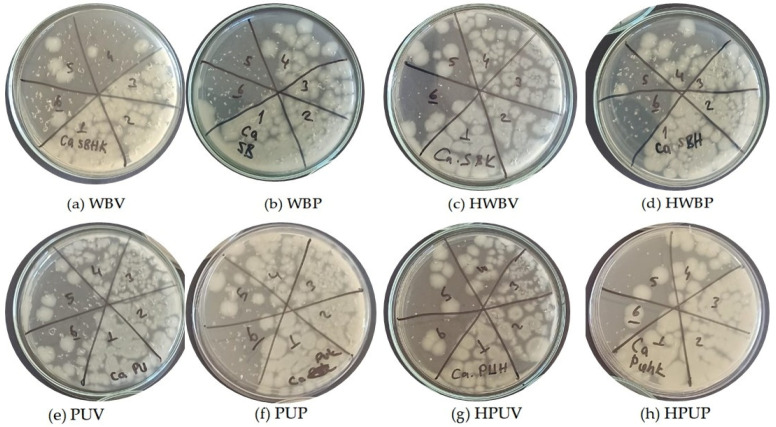
Images of colony development from *C. albicans* dilutions inoculated onto MHA medium after UV treatment of specimens.

Figure 8 presents the specimens coded as (a) WBV, (b) WBP, (c) HWBV, (d) HWBP, (e) PUV, (f) PUP, (g) HPUV, and (h) HPUP after UV treatment. Images of colony development obtained from *C. albicans* dilutions inoculated onto MHA medium are shown. In the figure, label 1 corresponds to a cell concentration of 10^6^ CFU/mL, whereas label 6 corresponds to a cell concentration of 10^1^ CFU/mL.

Evaluation of the results for the yeast species *C. albicans* showed that UV aging of water-based coating systems generally increased antifungal activity. In particular, the WBV specimens, which do not contain any hygienic additives, exhibited a new antifungal activity of 70.5% after UV aging. This observation suggests that UV irradiation may enhance surface–microorganism interactions by promoting the formation of oxygen-containing functional groups on the coating surface, thereby increasing antifungal effectiveness [31].

For the HWBP specimen, antifungal activity increased from 90% to 93.69% after UV aging, indicating a limited but positive improvement. In contrast, antifungal activity was completely eliminated after UV aging in the HWBV and PUV systems, suggesting that UV exposure may induce photochemical degradation within both the coating matrix and the active antifungal components.

In contrast, antifungal activity in the HPUP specimens decreased from 96.8% to 20.6% after UV aging, corresponding to an approximately 76% loss in efficacy. This result suggests that polyurethane-based coating systems may be more susceptible to UV-induced photodegradation, particularly with respect to antifungal stability, as also reported for UV-sensitive polymer systems [45].

The observed changes in antimicrobial performance can be interpreted through two main processes: activation and degradation. UV irradiation can induce oxidative modifications on the coating surface, leading to the formation of oxygen-containing functional groups, such as hydroxyl and carbonyl groups, and, in some systems, ROS. These changes may increase surface reactivity and enhance the interaction between active components and microorganisms, thereby enhancing their interaction with microorganisms [31]. This mechanism explains the improved antimicrobial performance observed in certain coating systems, particularly in waterborne coatings with more flexible matrix structures (e.g., HWBV).

On the other hand, UV exposure can lead to the degradation of both the polymer matrix and antimicrobial additives, resulting in the breakdown or deactivation of active compounds and consequently reducing or eliminating antimicrobial performance, particularly in systems with low photostability [45,48].

This degradation process is more pronounced in highly crosslinked and dense polyurethane-based systems (e.g., HPUV), where the mobility of active components is inherently limited. As a result, active agents cannot effectively reach the surface, and their interaction with microorganisms is restricted. When combined with UV-induced photodegradation, this leads to a complete loss of antimicrobial activity [31].

These processes help explain the different behaviors observed among the coating systems in this study. For example, the improved antimicrobial activity observed in HWBV coatings after UV aging can be attributed to the greater mobility of active agents within the more flexible waterborne matrix. In contrast, the complete loss of antimicrobial activity in HPUV coatings is likely due to the photodegradation of active components combined with their restricted mobility within the highly crosslinked polyurethane network.

Similarly, the absence of antimicrobial activity in PUV and HPUP coatings both before and after UV aging can be explained by the immobilization of active agents within the dense polyurethane matrix, which limits their availability at the surface and prevents effective interaction with microorganisms [46,47]. In addition, the lack of photocatalytic or UV-activatable components in these systems may further hinder the development of antimicrobial activity under UV exposure. Consequently, the active components remain embedded within the coating matrix and cannot exert their antimicrobial effect at the surface [30,49].

In coating systems containing nanopigments and antimicrobial additives, UV aging also affects color stability and antimicrobial performance through closely related mechanisms. Nanopigments can act as UV absorbers or scattering agents, protecting the polymer matrix from photodegradation and preserving both optical and functional properties [50]. In such cases, improved color stability is often associated with sustained or enhanced antimicrobial activity. This relationship is particularly important in coatings such as HWBV, in which the relatively low color variation observed after UV aging coincided with improved antimicrobial performance. This suggests a possible synergistic effect between UV shielding and the preservation of active components, contributing to the maintenance of both aesthetic and biological properties.

However, if nanopigments or antimicrobial additives lack sufficient photostability, UV exposure may induce their degradation or promote their migration away from the surface. This may lead to increased color change (higher ΔE values) accompanied by a simultaneous reduction in antimicrobial performance [45,48]. Accordingly, the relationship between color stability and antimicrobial performance should be interpreted as a coupled response controlled by additive photostability, dispersion within the matrix, and compatibility with the coating system.

### 3.2. Results of the Color Measurement Tests

One-way analysis of variance (ANOVA) general linear model procedures were performed separately on the color measurement data (∆L*, ∆a*, ∆b*, ∆E*) from the specimens to analyze the effect of protective surface coating materials on mean color measurements. The ANOVA results for the color measurements are presented in Table 5.

The results of the ANOVA performed on the color-change parameters (∆L*, ∆a*, ∆b*, ∆E*) of the test specimens with protective coatings indicate statistically significant differences for all parameters (*p* < 0.005). ANOVA results also revealed that the interactions between protective coatings and ∆L*, ∆a*, ∆b*, and ∆E* were statistically significant (*p* < 0.05). To further identify the sources of these differences, homogeneity-group tests and post hoc multiple-comparison analyses were conducted to allow pairwise evaluation of the effects of different coating systems on color changes after UV aging.

The ∆L*, ∆a*, ∆b*, and ∆E*, values obtained before and after UV aging for the coated test specimens are presented in Table 6.

According to Table 6, the lowest ∆L* values under UV aging conditions were observed in the HPUV, PUP, HPUP, WBP, and HWBP coatings, which belong to the same homogeneity group; these coatings were therefore more resistant to lightness changes than the PUV, WBV, and HWBV coatings. The negative change in L* values for all coating types indicates surface darkening, with the greatest darkening observed in the HWBV coating (−6.51).

It is known that polyurethane-based coatings form more stable films against photodegradation due to their highly crosslinked polymer network structures [3,51]. Accordingly, the relatively low ∆L* values observed in polyurethane-based systems can be attributed to their enhanced structural stability. Furthermore, the UV-absorbing and radical-scavenging effects of hygienic additives may have contributed to maintaining lower ∆L* values by limiting photooxidative reactions within the polymer matrix [24].

The literature reports that in uncoated beech specimens, the ΔL value reached −20.90 after 168 h of UV exposure [52], whereas in coated specimens it reached −14.05 after 200 h [53]. The lower ΔL values obtained in this study can be attributed to both the protective role of the coating systems and the shorter exposure duration.

When the chromatic axes were examined, the smallest changes in ∆a* and ∆b* values were observed in the HWBV coating, indicating that this coating is more effective in maintaining chromatic stability. It has been reported that acrylic/polyurethane dispersions used in waterborne coatings, when supported with UV stabilizers, can reduce color deviations [9,54]. In addition, the homogeneous distribution of hygienic additives within the binder matrix may have contributed to this limited chromatic variation.

A positive shift in the a* parameter was observed for all coatings, indicating a tendency toward red tones. The highest increase was observed for the WBP coating, while HPUP, PUP, and HWBP coatings showed high levels of change; PUV and WBV showed moderate changes; and HPUV and HWBV showed relatively limited variation. These results indicate that coating chemistry and film formation characteristics play a key role in chromatic response.

Similarly, positive changes were observed in the b* parameter across all coatings, indicating a general tendency toward yellowing. The highest increase was observed for the WBP coating, whereas HPUP and several other coatings exhibited moderate-to-high levels of yellowing. The lowest ∆b* was observed for the HWBV coating. These findings emphasize the influence of binder structure and film properties on color behavior along the yellow–blue axis.

The lowest ∆E* value was obtained for the HPUV coating (6.12), indicating that this system best preserved color integrity after UV aging. The literature suggests that ∆E* values in the range of 6–7 correspond to perceptible but acceptable color changes [9,54]. This result implies that polyurethane varnishes modified with hygienic additives may offer aesthetic advantages for interior applications.

In contrast, the highest ∆E* value was observed in the WBP coating, indicating substantial color alteration [55]. HPUP, PUV, PUP and HWBP coatings exhibited pronounced color changes, whereas WBV showed moderate color variation. Relatively low ∆E values were maintained in the HPUV and HWBV coatings, indicating better color stability. These differences highlight the role of coating composition, pigment content, and film formation properties in determining optical performance.

When considering perceptual threshold values, all coating systems resulted in noticeable color changes. In particular, WBP and HPUP coatings caused significant visual alterations, whereas PUV, PUP, and HWBP coatings were at the threshold of a strong color change. WBV, HPUV, and HWBV coatings exhibited comparatively low but still perceptible optical variations.

## 4. Conclusions

The findings of this study reveal that the UV aging process has a decisive effect on both the biological and aesthetic performance of coating systems applied to wood surfaces. UV aging exhibited system-dependent effects, with certain waterborne coating systems maintaining or enhancing antimicrobial efficacy, whereas polyurethane-based systems more frequently exhibited loss of activity due to photodegradation. Notably, waterborne varnish systems demonstrated a more stable antimicrobial response than other coating types. For instance, the highest antibacterial activity against *E. coli* was observed in the HWBP system after UV aging, representing a reduction of up to 96.2%, whereas no activity was detected in the PUV and HPUP systems.

In coatings containing hygienic additives, the effect of UV irradiation varies depending on the chemical structure of the additive and its interaction with the binder matrix. UV exposure may enhance antimicrobial performance in some systems by facilitating the migration or activation of active components at the surface, whereas in others, it may induce photodegradation of these components, leading to reduced effectiveness. For example, antifungal activity in the WBV system increased from 0% to approximately 70.5% after UV exposure, whereas a complete loss of activity was observed in the PUV and HWBV systems.

When evaluated by microorganism type, UV aging generally exhibited an efficacy-enhancing effect against Gram-negative bacteria *(E. coli) and* yeast (*C. albicans*), whereas a more complex, coating-dependent behavior was observed for Gram-positive bacteria *(S. aureus*). In particular, antimicrobial activity against *S. aureus* increased by up to 80% in HWBV coatings, whereas it completely disappeared in HPUV systems. This variation is likely attributable to differences in microbial cell wall structures and their interactions with UV-induced physicochemical modifications on coating surfaces.

From an aesthetic perspective, UV aging significantly affected the color parameters of all coating systems. In water-based systems, particularly waterborne varnish formulations, low ∆a* and ∆b* values indicate superior chromatic stability. In polyurethane-based systems, the relatively low ∆L* and ∆E* values observed in certain specimens suggest that the binder matrix provides resistance to changes in lightness owing to its stable network structure. The lowest ∆E* was observed in HPUV coatings (6.12), whereas the highest was observed in WBP coatings (18.34), indicating a substantial visual alteration. However, the extent of ∆E* was primarily governed by coating composition, the presence of pigments, and the photostability of incorporated additives. These results indicate that coating composition governs not only protective performance but also aesthetic durability, with ∆E* serving as a key indicator of color stability.

Overall, the results clearly demonstrate that UV aging is a critical factor influencing both antimicrobial performance and color stability, and that these effects vary significantly depending on coating type and formulation. The findings further indicate that waterborne varnish systems modified with hygienic additives provide a more balanced performance in terms of maintaining antimicrobial activity and color stability under UV exposure. These systems achieved both high antimicrobial performance and relatively low color change compared to other coating types.

This study contributes to the literature by highlighting the importance of evaluating biological and aesthetic performance simultaneously under UV-aging conditions. In the design of hygienic coatings for wood surfaces exposed to UV radiation, particular attention should be paid to the photostability of active components, their interaction with the binder matrix, and their ability to minimize color changes. These parameters should be considered key design criteria for the development of durable, functional, and application-oriented coating systems. These systems achieved both high antimicrobial performance and relatively low color change compared to other coating types.

## Figures and Tables

**Table 1:** Some properties of the coating materials used in the study [17,39].

Group	Protective Coating Material	Solids Content (%)	Coating Thickness (µm)	Property
1	WBV	29.56	19.40	Waterborne acrylic resin
2	HWBV	29.35	22.10	Ag-modified waterborne acrylic resin
3	WBP	29.41	21.20	Nano pigment (Np)-modified waterborne acrylic resin
4	HWBP	29.81	22.40	Ab- and Np-modified waterborne acrylic resin
5	PUV	39.80	25.20	Solvent-borne polyurethane resin
6	HPUV	41.60	26.50	Ag-modified solvent-borne polyurethane resin
7	PUP	40.39	25.10	Np-modified solvent-borne polyurethane resin
8	HPUP	41.92	26.40	Ag- and Np-modified solvent-borne polyurethane resin

**Table 2:** Logarithmic and percentage comparisons of the antimicrobial activities of wood specimens against *E. coli* before and after UV treatment.

	Aging Process				
Protective Coating	Control Log _10_ Reduction	Control % Reduction	UV 24 h Log _10_ Reduction	UV 24 h % Reduction	UV 24 h Change
WBV	0	0	0	0	No change.
WBP	0.61	75%	0.22	39.8%	Antimicrobial activity decreased.
HWBV	0	0	0.59	74.3%	Antimicrobial activity a new increased.
HWBP	1.0	90%	1.42	96.2%	Antimicrobial activity increased.
PUV	0	0	0	0	No change.
PUP	0	0	0.52	69.8%	Antimicrobial activity a new increased.
HPUV	0.13	26%	0	0	Antimicrobial activity decreased.
HPUP	0	0	0	0	No change.

**Table 3:** Antimicrobial activities of wood specimens against *S. aureus* before and after UV treatment.

	Aging Process				
Protective Coating	Control Log _10_ Reduction	Control % Reduction	UV 24 h Log _10_ Reduction	UV 24 h % Reduction	UV 24 h Change
WBV	0	0	0	0	No change
WBP	0	0	0	0	No change
HWBV	0	0	0.7	80.0%	Antimicrobial activity increased.
HWBP	0.04	8.8%	0	0	Antimicrobial activity decreased.
PUV	0.15	29.2%	0.52	69.8%	Antimicrobial activity increased.
PUP	0.04	8.8%	0.46	65.3%	Antimicrobial activity increased.
HPUV	0.09	18.7%	0	0	Antimicrobial activity decreased.
HPUP	0	0	0	0	No change

**Table 4:** Logarithmic and percentage comparisons of the antimicrobial activities of wood specimens against *C. albicans* before and after UV treatment.

	Aging Process				
Protective Coating	Control Log _10_ Reduction	Control % Reduction	UV 24 h Log _10_ Reduction	UV 24 h % Reduction	UV 24 h Change
WBV	0	0	0.53	70.49%	Antimicrobial activity increased.
WBP	0	0	0	0	No change
HWBV	0.26	45.05%	0	0	Activity has completely disappeared.
HWBP	1	90.00%	1.2	93.69%	Limited increase in activity (20%)
PUV	1.3	94.99%	0	0	Activity has completely disappeared.
PUP	0	0	0	0	No activity.
HPUV	0	0	0	0	No activity.
HPUP	1.5	96.84%	0.1	20.57%	Activity has decreased significantly.

**Table 5:** ANOVA results of the test specimens for each color parameter.

	Source	Sum of Squares	Degrees of Freedom	Mean Square	F-Value	Significance
∆L*	Between Groups	266,201	7	38,029	16,926	0.000
	Within Groups	161,769	72	2247		
	Total	427,970	79			
∆a*	Between Groups	1,219,612	7	174,230	118,623	0.000
	Within Groups	105,752	72	1469		
	Total	1,325,363	79			
∆b*	Between Groups	830,347	7	118,621	6399	0.000
	Within Groups	1,334,671	72	18,537		
	Total	2,165,018	79			
∆E*	Between Groups	1,014,752	7	144,965	21,533	0.000
	Within Groups	484,717	72	6732		
	Total	1,499,470	79			

**Table 6:** Homogeneity group test to identify the group or groups causing differences obtained from the interactions of (∆L*), (∆a*), (∆b*), and (∆E*) with the protective coating.

		∆L*		∆a*		∆b*		∆E*	
Protective Coatings	N	$\bar{X}$ (SD)	HG	$\bar{X}$ (SD)	HG	$\bar{X}$ (SD)	HG	$\bar{X}$ (SD)	HG
PUV	10	−4.97 (2.26)	B	3.48 (1.23)	C	6.10 (10.61)	BCD	12.74 (4.61)	BC
HPUV	10	−1.34 (2.28)	C	2.05 (1.27)	D	4.89 (2.17)	CD	6.12 (1.77)	D
PUP	10	−1.44 (0.59)	C	9.76 (1.17)	B	7.36 (2.21)	BC	12.36 (2.25)	BC
HPUP	10	−2.10 (0.30)	C	10.24 (0.73)	B	10.15 (1.19)	B	14.58 (1.27)	B
WBV	10	−4.45 (1.74)	B	2.93 (0.85)	C	8.63 (1.23)	BC	10.33 (1.01)	C
HWBV	10	−6.51 * (1.89)	A	1.86 (0.99)	D	2.82 (3.05)	D	7.91 (2.01)	D
WBP	10	−2.29 (0.44)	C	11.44 * (2.08)	A	14.14 * (3.44)	A	18.35 * (3.95)	A
HWBP	10	1.62 (0.59)	C	9.62 (0.76)	B	7.18 (1.34)	BC	12.14 (1.39)	BC
LSD		1.48		1.20		4.28		2.58	

$\bar{X}$: Arithmetic mean, SD: Standard deviations, HG: Homogeneity group, *: Highest value.

## Data Availability

The original contributions presented in this study are included in the article. Further inquiries can be directed to the corresponding author.

## Abbreviations

The following abbreviations are used in this manuscript:

WBV	Waterborne varnish
HWBV	Ag-modified waterborne varnish
WBP	Nanopigment-modified waterborne paint
HWBP	Ag-modified and nanopigment-modified waterborne paint
PUV	Solvent-borne polyurethane varnish
HPUV	Ag-modified solvent-borne polyurethane varnish
PUP	Nanopigment-modified solvent-based polyurethane paint
HPUP	Ag-modified and nanopigment-modified polyurethane paint
Ag	Silver
*E. coli*	*Escherichia coli*
*S. aureus*	*Staphylococcus aureus*
*C. albicans*	*Candida albicans*
ΔL*	Lightness difference
Δa*	Red–green color difference
Δb*	Yellow–blue color difference
ΔE*	Total color difference
LSD	Least Significant Difference
ROS	Reactive oxygen species
UV	Ultraviolet
CFU/mL	Colony-forming units per milliliter
CIE	International Commission on Illumination
